# Genome sequence of a lytic phage phi1_092060 targeting ST2 KL104-type *Acinetobacter baumannii*

**DOI:** 10.1128/mra.00156-25

**Published:** 2025-04-15

**Authors:** Li Wei, Jiayuan Qin, Yu Feng, Zhiyong Zong

**Affiliations:** 1 Center of Infectious Diseases, West China Hospital, Sichuan University, Chengdu, China; 2 Department of Infection Control, West China Hospital, Sichuan University, Chengdu, China; 3 Division of Infectious Diseases, State Key Laboratory of Biotherapy, Chengdu, China; 4 Center for Pathogen Research, West China Hospital, Sichuan University, Chengdu, China; Department of Biology, Queens College, Queens, New York, USA

**Keywords:** *Acinetobacter baumannii*, carbapenem resistance, capsular polysaccharide, phage, phage therapy

## Abstract

We describe the genome of a lytic phage isolated from sewage, which is capable of lysing ST2 KL104-type carbapenem-resistant *Acinetobacter baumannii* strains. The genome is 167,208 bp in length, has a guanine-cytosine (GC) content of 37%, and includes 266 protein-coding sequences and five tRNAs.

## ANNOUNCEMENT

Carbapenem-resistant *Acinetobacter baumannii* (CRAb) has been designated by the World Health Organization as a critical pathogen of concern (1). Treatment options for CRAb are severely limited (2, 3), making phage therapy a promising alternative (4). While phages targeting sequence type 2 (ST2) and capsular type KL2 CRAb have been reported (5–7), no phages specific to ST2 KL104 CRAb have been described. Expanding the library of lytic phages for precise targeting of specific strain types is of significant clinical and practical importance. Here, we present the genome of phi1_092060, a phage capable of effectively lysing ST2 KL104 CRAb clinical isolates.

In July 2024, phi1_092060 was isolated from untreated sewage at the wastewater treatment plant of West China Hospital using the CRAb clinical strain 091006 (accession no. JBLLSI000000000), following the methodology described in previous studies (8). Strain 091006 belongs to sequence type 2 and capsular type KL104. Individual phage plaques were purified three times to ensure phage purity. Genomic DNA of phi1_092060 was extracted from purified phage particles using the Phage DNA Isolation Kit (Norgen Biotek, Thorold, Canada) according to the manufacturer’s instructions. Sequencing libraries were prepared using the NEBNext Ultra II DNA Library Prep Kit (New England Biolabs, Ipswich, MA, USA), and sequencing was performed on the HiSeq X10 platform. Raw sequencing reads underwent quality control with Trimmomatic v0.39 (9) to remove adapter sequences and discard reads shorter than 130 bases. Genome assembly was performed using Unicycler v0.5.0 (10). Contamination was assessed using CheckV, and non-phage contigs were discarded (11). Genome annotation was conducted using Pharokka v1.7.2 (12), and BLAST was used to identify the phage with the highest overall DNA similarity (identity × coverage) (13). Unless specified otherwise, the software was run with default parameters.

The genome sequencing of phi1_092060 produced 4,217,862 pairs of 150 bp reads, totaling 1.27 Gb (accession no. SRR32014619). The genome of phi1_092060 is 167,208 bp in length, with a GC content of 37%, 266 predicted coding sequences (CDSs), and five tRNAs. BLAST analysis showed that phi1_092060 shares the highest DNA similarity (96.38%, identity × coverage) with *Acinetobacter* phage AB-Navy97 (accession no. OL770261.1), which belongs to the genus *Lazarusvirus* of the family *Straboviridae* within the class *Caudoviricetes*.

**Fig 1:**
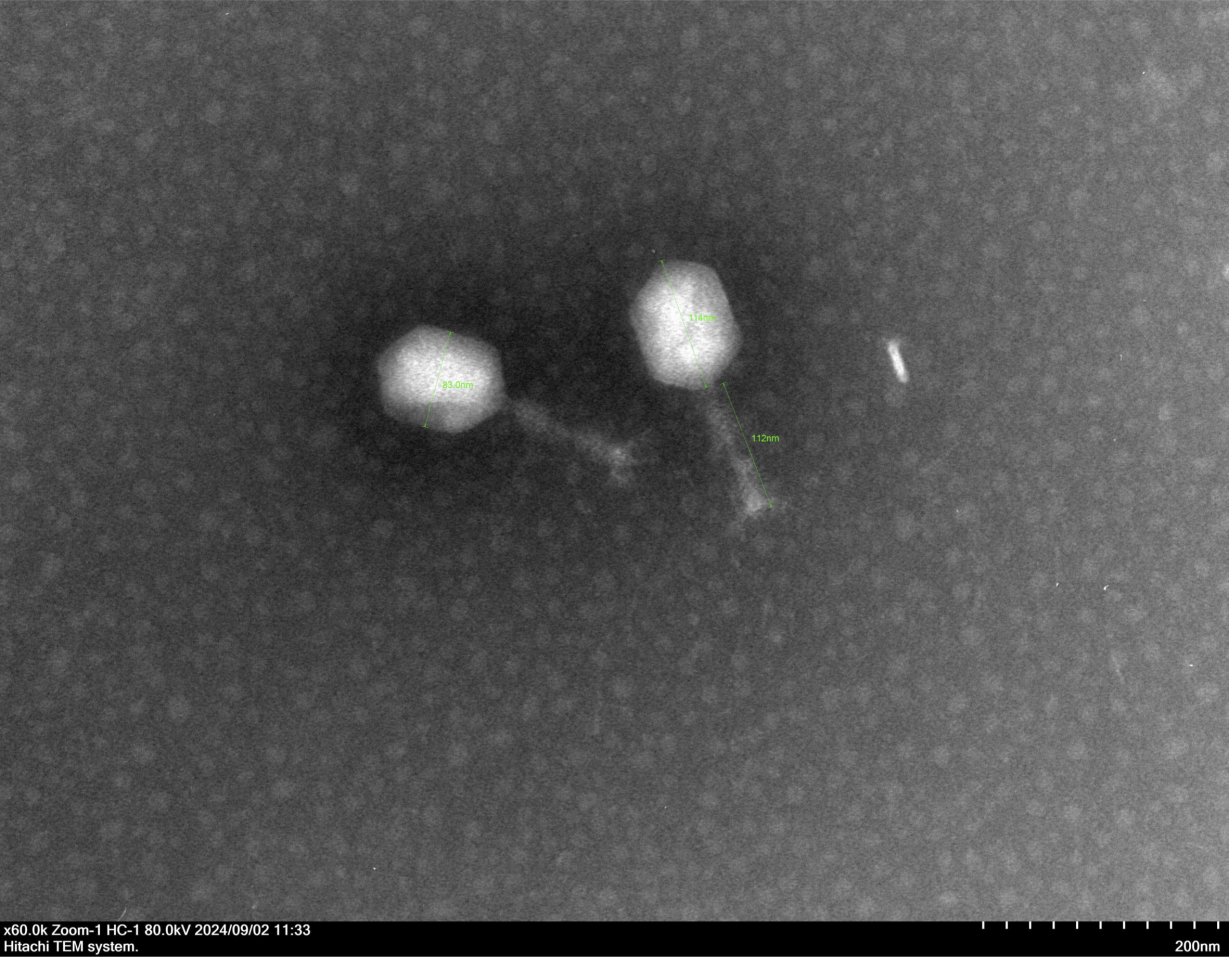
The morphology of phage phi1_092060 under transmission electron microscopy. The scale was 200 nm.

Negative staining of the phi1_092060 phage particles was performed using 2.0% (wt/vol) uranyl acetate. The morphology of phi1_092060 was observed under a Hitachi transmission electron microscope (Hitachi, Japan) at an accelerating voltage of 80 kV. phi1_092060 features a polyhedral head structure, approximately 83 nm in diameter, and a long, rod-shaped, contractile tail approximately 112 nm in length (Fig. 1). The host range of phi1_092060 was determined as described previously (14). phi1_092060 was able to lyse ST2-KL52, ST2-KL104, and ST195-KL45 CRAb isolates. No known antimicrobial resistance or virulence genes were identified in the phi1_092060 genome using the CARD v4.0.0 (15) and VFDB 2025 (16) databases. The high BACPHLIP (17) score (83.7%) suggested a virulent lifestyle for phi1_092060. As such, phi1_092060 meets the standards for clinical phage therapy applications, thereby expanding the phage arsenal against difficult-to-treat CRAb pathogens.

## Data Availability

The complete genome sequence of *Acinetobacter baumannii* phage phi1_092060 has been deposited in GenBank under accession number PQ885451.1 and Sequence Read Archive (SRA) accession number SRR32014619. The version described in this paper is the first version.
